# A movement for movement: an exploratory study of primary healthcare professionals’ perspectives on implementing the Royal College of General Practitioners’ active practice charter initiative

**DOI:** 10.1186/s12875-024-02345-0

**Published:** 2024-04-15

**Authors:** Callum J. Leese, Robert H. Mann, Hussain Al-Zubaidi, Emma J. Cockcroft

**Affiliations:** 1 Department of Population Health and Genomics, Ninewells Hospital, University of Dundee, James Arnott Drive, Dundee, United Kingdom; 2 Department of Public Health and Sport Sciences, Faculty of Health and Life Sciences, University of Exeter, Exeter, United Kingdom; 3 Physical Activity and Lifestyle Champion, Royal College of General Practitioners, London, United Kingdom; 4 Department of Health and Community Sciences, Faculty of Health and Life Sciences, Royal College of General Practitioners, Exeter, United Kingdom

**Keywords:** Physical activity, Primary health care, General practice, Health promotion, Behaviour change, Social prescribing

## Abstract

**Background:**

Regular physical activity (PA) results in extensive physical, psychological, and social benefits. Despite primary care being a key point of influence for PA behaviours in the UK, research indicates poor application of PA interventions in this context. To address this, the Royal College of General Practitioners’ (RCGP) developed and implemented the Active Practice Charter (APC). The aim of the study was to evaluate the perceived impact and acceptability of the APC initiative from the perspective of primary healthcare professionals (PHPs).

**Methods:**

An online exploratory cross-sectional survey was designed to assess the perceived impact, experiences, and challenges of the APC initiative, from the perspective of PHPs. The survey was distributed by the RCGP via email to 184 registered APC practices across the UK.

**Results:**

Responses were reviewed from staff (*n* = 33) from 21 APC practices. Initiatives used by APC practices included: educational programmes, partnerships with PA providers, referral systems, and infrastructure investment. Perceived benefits included: increased awareness about PA, staff cohesion, and improved well-being. However, staff felt the APC had limited effect due to implementation barriers, including: a lack of engagement, time, resources, and funding.

**Conclusion:**

This is the first evaluation of any nationwide UK-based initiative engaging GP practices in promoting PA. Acknowledging the limitations in response rate, although support exists for the RCGP APC, the evaluation highlights challenges to its implementation. Nonetheless, the wide reach of the RCGP, combined with the cited staff and patient benefits, demonstrates the significant potential of the APC initiative. Given the need to address physical inactivity nationally, further development the APC offers a possible solution, with further research required to overcome the challenges to implementation.

**Supplementary Information:**

The online version contains supplementary material available at 10.1186/s12875-024-02345-0.

## Background

Research demonstrates that regular physical activity (PA) results in extensive physical, psychological, and social benefits [1]. In 2019 the Chief Medical Officers for the UK introduced updated PA guidelines, recommending that adults aim to accumulate at least 150 min of moderate-intensity aerobic PA per week, including at least two sessions per week aimed at muscle strength and balance [2]. These guidelines also recommend minimising sedentary behaviour. Despite this guidance, one third of adults in the UK regularly fail to meet the Chief Medical Officers’ PA guidelines [3], having huge implications on an already stretched health service. For example, according to the Department of Health and Social Care, physical inactivity is associated with 1 in 6 deaths in the UK and costs the NHS £0.9 billion annually (and £7.2 billion to the UK economy) [4].

Primary healthcare professionals (PHPs) have wider exposure to the whole population than any other health professional – regularly seeing those in need of PA advice and viewed by the public as a trusted source of information [5, 6]. Given this level of exposure, it is not surprising that lifestyle interventions delivered via primary care have been shown to be effective at initiating behavioural change and reducing the risk of disease progression [7]. Research has also shown that PA interventions delivered in primary care are effective at increasing PA in patients [8] and are cost-effective [9]. This supports the shifting emphasis in healthcare settings from treatment to prevention, partially as a consequence of the increasing burden of non-communicable diseases [10]. Acknowledging this, World Health Organisation Europe highlighted PA counselling in primary care as one of its ‘best buys’ in an economic analysis of cost per disability-adjusted life years averted [11].

Despite primary care being a key point of influence for PA behaviours in the UK (and globally), evidence points to poor implementation of PA promotion by General Practitioners (GPs) [12, 13]. PHPs frequently cite four reasons for this: (1) lack of time; (2) insufficient knowledge and skills; (3) a lack of resources and/or support; and (4) negative financial implications [14].

To address these challenges, the Royal College of General Practitioners (RCGP), with support from Sport England, launched the Active Practice Charter (APC) initiative in 2018. To become a charter marked “Active Practice,” practices need to demonstrate that they have taken steps to: (1) increase PA and reduce sedentary behaviour in both patients and staff, and (2) partner with a local PA provider to support getting more people active. The RCGP have provided resources, support, and training to help practices achieve the APC accreditation via the *physical activity and lifestyle toolkit* [15]. A member of staff is employed on a part-time basis to support practices in achieving the accreditation, which can be used by the practice to evidence development during future revalidation and commissioning reviews. To date, almost 200 practices have been awarded the APC, with ongoing evolution and expansion.

The aim of the present exploratory evaluation was to assess the perceived impact, experiences, and challenges of the APC initiative from the perspective of PHPs working in charter marked “Active Practices.”

## Methods

### Survey design and distribution

The exploratory survey was developed by an advisory panel including RCGP representatives (*n* = 2), academics (*n* = 2), and GPs with a special interest in PA (*n* = 3). The mixed-methods survey included a mixture of Likert scales, closed questions, and free-text response questions (see Supplementary File 1). Once developed, an online version of the survey was created using the Jisc Online Survey (JOS) tool.

Specifically, the survey was designed to assess: (1) demographics and (2) the perceived impact of the APC. Free-text response questions were used to assess the perceived impact of the APC on: (1) healthcare delivery; (2) the experiences of APC for staff; and (3) the challenges related to APC implementation. Response to questions about “experiences” and “challenges” were broadly related to the acceptability of the APC for PHPs. The survey comprised of 12 questions of which seven were compulsory.

The survey was distributed via email by an RCGP Senior Project Manager to the contacts registered in the RCGP database for all practices that had been awarded the APC. This represented 184 practices at the date of distribution (18/08/2022). At least one staff representative from each practice was asked to complete the survey. A reminder email was sent by the same RCGP Senior Project Manager at both 4- and 8-weeks following initial contact, with the survey closing after 10 weeks (27/09/2022). The survey was also advertised at two national events for PHPs during this 10-week data collection period. Participation was voluntary and unpaid, with completion of the survey via computer and/or smartphone.

### Participants

Only employed staff (both clinical and non-clinical) of accredited APC practices in the UK were invited to participate. Responses from practices that had not been awarded the APC were ineligible, with any responses from these practices deleted.

### Data analysis

Data was downloaded and cleaned in Excel before importing into IBM SPSS (version 28.0.0.0). Descriptive statistics were used to present demographics data, closed questions, and Likert-scaling questions.

Survey responses for the open-ended qualitative questions were analysed using a content analysis as described by Hsieh and Shannon [16]. A content analysis is an inductive method of research, used to identify patterns within qualitative data in a systematic way. An inductive method of analysis was selected to allow for identification of themes in a field with limited pre-existing research, with a content analysis used to allow for systematic coding and categorisation. Free text responses were imported into NVivo (version 12) for analysis. The analysis involved the following five stages: (a) all free text responses were read by one author (CL) in order to ensure familiarisation with the data; (b) data was divided into three subordinated themes (perceived impact, experiences of the APC initiative and challenges to implementation) identified at the survey creation and directed by the research question; (c) the free text was analysed line by line and coded into sub-categories; (d) generated codes were categorised into themes according to similarities and differences; (e) a frequency analysis of generated themes was conducted to explore whether certain challenges were experienced more frequently than others. Data analysis was primarily conducted by one author (CL), with discussion with additional authors (EC and RM) at key stages.

### Ethics

The project was identified as a quality improvement project and, as a result, ethics was waived by the Tayside Medical Science Centre Research Board. The project was registered with NHS Tayside Clinical Governance Team (032/22).

## Results

### Demographics

At time of evaluation, 184 practices in the UK had been awarded the APC. Of these practices, 33 staff responded to the survey, from 21 different practices (11%). All the submitted responses were fully complete and included within the analysis. Of the respondents, 51.5% (*n* = 17) were practice administration personnel, 36.4% (*n* = 12) were GPs, and the remainder comprised of coaches, dispensers, paramedics, or practice nurses. After removing duplicate responses from practices, 57.1% (*n* = 12) had a patient list size greater than 10,000.

Of the 21 individual practices that responded, 16 (76.2%) had partnered with a local parkrun, and six (28.6%) had partnered with a community walking group. Four (19%) had partnered with other organisations, such as RunTalkRun and Walk for Health, with seven (33.3%) of the practices partnering with more than one PA provider.

### Results of thematic analysis

A summary of the qualitative content analysis with description of themes is provided in Table 1. As identified in the analysis plan, an inductive method was used following the development of three themes identified from project design: experiences of APC initiative, perceived impact of the APC initiative, and challenges to implementation. Results have been presented below under each of these three headings.

**Table 1:** A summary of the qualitative content analysis for the survey assessing the perceived impact, experiences, and challenges of the APC initiative

Themes relating to experience of APC.			
Theme	Theme Description	Percentage of Total Respondents (*n* = 33)	Illustrative Quote(s)
Education	Practices share educational information with their patients about the benefits of physical activity	69.8%	*“As a surgery we are always promoting the benefits of being active, this is shared with our patients through social prescribing and also through our social media platforms.”* *“There is a lot more information on both staff and patient notice boards about ways of getting more active and a lot of promotion within the staff”*
Engagement with Third Party Providers	Collaboration between practices and PA providers to promote and deliver physical activity, for example parkrun	66.7%	*“we have great relationships with active partnerships locally and are building on this to bring in more opportunities for pts”* *“We are able to suggest local activities available to patients looking to increase activity and encourage this”*
Referral Systems	Healthcare professionals can refer patients to non-medical staff specifically trained in delivering physical activity promotion	33.3%	*“GP referral and self referral schemes”* *“Social prescribing for local outdoor activities and Nordic walking”*
Participation in staff-based PA challenges	Many practices adopted time-limited physical activity challenges to promote staff engagement and cohesion, for example a combined practice team virtual walk from Lands End to John O’Groats.	24.2%	*“Staff members have taken on active challenges they were thrilled to achieve ie Keswick to Barrow 40 mile walk”* *“Step challenge, Lands End to John O Groats virtual walk on strava 5 k Race for life as a practice”*
Investment in infrastructure	Practices looked to invest in aids to promote physical activity and decrease sedentary behaviour. These included standing desks and bike lock-up areas.	18.2%	*“I’d love to hear ideas from other practices and what they’ve done to make changes. I love the idea of putting a watt/spin type bike in the waiting room, but the idea of cleaning/wiping down/safety measures could outweigh the actual use of it.”* *“using standing desks…to reduce sedentary behaviour”*
**Themes relating to perceived impact of APC.**			
Positive impact health and wellbeing	The APC has been perceived to have a positive impact at improving patients and staffs PA, decreasing SB and improving wellbeing	66.6%	“*Patients are getting involved in local community activities that we advertise in the waiting room, staff are more conscious of their own activity levels - using standing desks more to reduce sedentary behaviour.”* *“This is a fabulous initiative and encourages us to consider exercise as a priority when seeing our patient population”* *“Helped resilience within the team*”
No clear impact	Respondents did not feel that the APC had positively impacted themselves, patients or staff. Many agreed with the idea but felt delivery needed reformed.	33.3%	*“What is it, other than a badge?”* *“I feel I need more in house support to deliver more effectively”*
**Themes relating to challenges of APC implementation**			
Engagement	Difficulty in persuading staff and patients to implement lifestyle changes to increase their personal PA and decrease their SB, and staff engagement in promotion of PA	45.5%	*“Keeping staff motivated to continue to reduce sedentary behaviour and increase activity when tired and have been busy”* *“it has been difficult to motivate patients to reduce sedentary behaviour”*
Time	Restrictions on personal time leading to lack of uptake of PA in both patients and staff, and restrictions on staff time limiting engagement in PA promotion	27.3%	*“Off the scale demand in general practice at the moment”* *“[patients] are so busy they don’t want to take it on”*
Financial implications	PA activities can lead to financial costs to patients, and infrastructure to encourage PA and decrease SB within medical centres can also be costly.	12.1%	*“Rurality with associated costs”* *“Equipment i.e. standing desks expensive”*

Abbreviations: APC: Active Practice Charter; PA: Physical Activity; GP: General Practitioner

### Perceived impact of the APC initiative

Approximately half (52%, *n* = 17) of staff that responded to the survey felt that being awarded the APC had improved their personal PA levels.

As shown in Table 2, respondents felt that being awarded the APC had improved staff PA levels (64% agreement, *n* = 21) and decreased staff sedentary behaviour (61% agreement, *n* = 20). Similarly, the majority of respondents felt that it had positively impacted patients, with increasing PA levels (52% agreement, *n* = 17). However, the reported impact on patient sedentary behaviour was less clear (52% of respondents neither agreed nor disagreed). As identified in Table 2, a small but noticeable number of respondents felt that the APC did not positively impact staff and patient physical activity or sedentary behaviour.

**Table 2:** Subjective assessment (via Likert scale) by staff of the impact of the Active Practice Charter on staff and patient physical activity and sedentary behaviour levels

Item	Strongly agree, n (%)	Agree, n (%)	Neither agree nor disagree, n (%)	Disagree, n (%)	Strongly disagree, n (%)
Becoming an ‘Active Practice’ has been effective at improving staff physical activity levels	7 (21%)	14 (42%)	10 (30%)	2 (6%)	0 (0%)
Becoming an ‘Active Practice’ has been effective at decreasing staff sedentary behaviour	8 (24%)	12 (36%)	9 (27%)	3 (9%)	1 (3%)
Becoming an ‘Active Practice’ has been effective at improving patient physical activity levels	5 (15%)	12 (36%)	14 (42%)	2 (6%)	0 (0%)
Becoming an ‘Active Practice’ has been effective at decreasing patient sedentary behaviour	5 (15%)	9 (27%)	17 (52%)	2 (6%)	0 (0%)

**Fig. 1:**
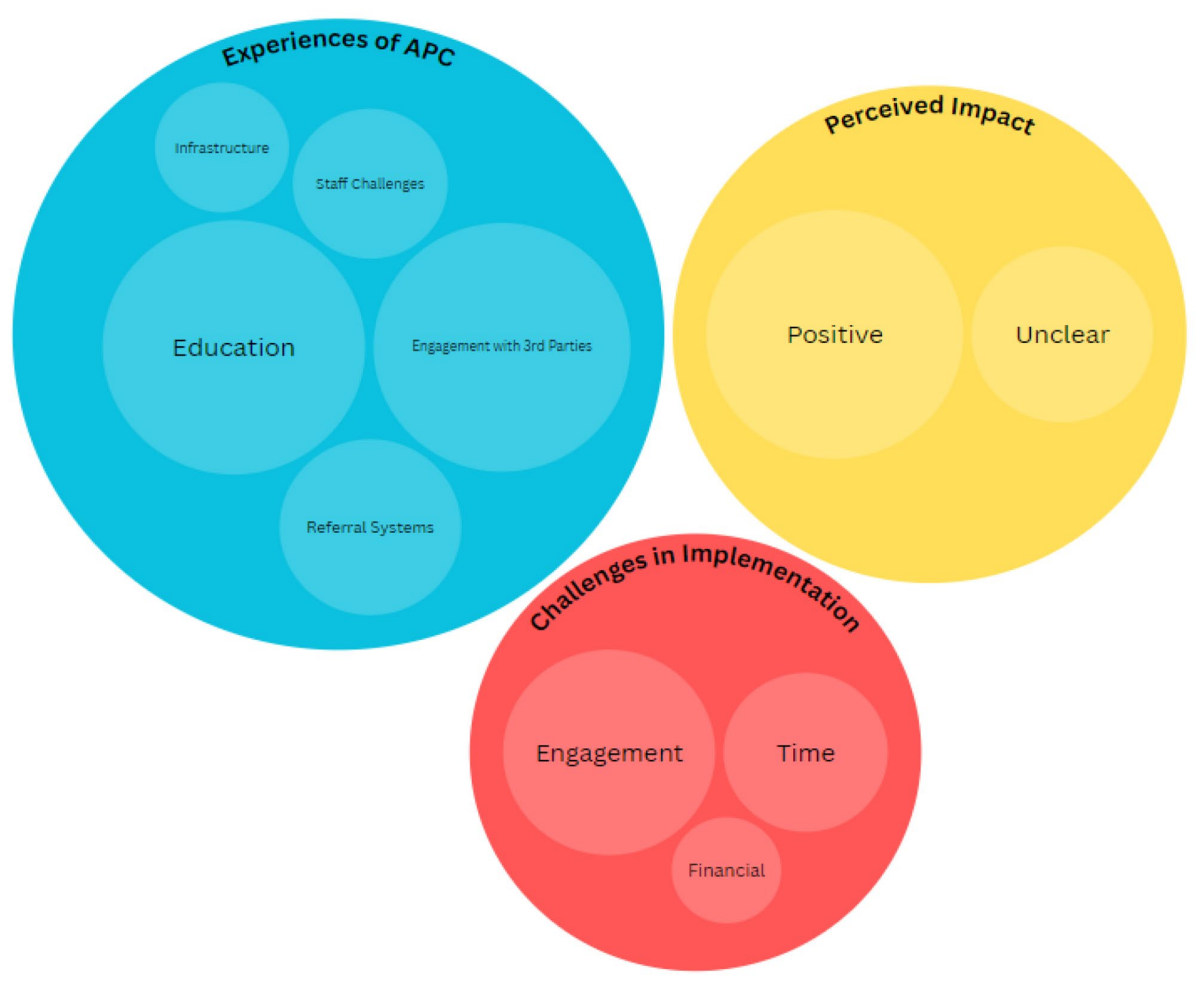
Predominant themes emerging from the qualitative thematic analysis, subdivided into (1) the perceived impact of the APC (2), the experience of the APC initiative and (3) the challenges to implementation. The graphic is weighted to represented frequency of response

The perceived impact of the APC was also explored via free-text responses, and is graphically represented in Fig. 1. Respondents reported a positive impact of the APC on: (1) increasing PA; (2) improved communication and education around the benefits of physical activity; (3) improved links within the local community; and (4) improved staff morale.

However, some respondents failed to see any benefit from the APC. For example, one respondent perceived the APC to be a ‘tick-box’ exercise:

“[…] what is it other than a badge?” (S5, GP).

### Experiences of the APC initiative

Responding practices identified ways in which the APC was implemented and experienced, through their free-text responses to the questions. These responses were categorised into five sub-themes: (1) education; (2) engagement with third party PA providers; (3) referral systems; (4) participation in staff-based PA challenges; and (5) investment in infrastructure.

The delivery of education, either to staff members or patients, was highlighted by 69.8% (*n* = 23) of respondents as a means for APC implementation. It commonly took the form of brief interventions with patients, however, the education of PHPs regarding the benefits of PA and the availability of resources was also reported.

Engagement with third party PA providers is required to achieve APC status and was described as an important part of the APC delivery with practices, with two-thirds (*n* = 22) of respondents highlighting the importance of engagement with third party PA providers as a method of APC implementation. Closely aligned to engagement with third party PA providers was referral systems, whereby PHPs can refer patients to non-medical staff trained in the delivery of PA promotion. One third of respondents (*n* = 11) highlighted this as a means of PA promotion through their experience of APC implementation.

Staff based PA challenges were also repeatedly attributed to the implementation of the APC, cited by 24.2% (*n* = 8) of respondents. The benefits of staff engagement through challenges were highlighted through one GP’s response:

“It helps to give patients an example that we are all trying. We can relate to their struggles and know what’s available locally” (S31, GP).

Finally, 18.2% (*n* = 6) of respondents highlighted that their practice looked to invest in tools to promote PA and decrease sedentary behaviour. These included standing desks and bike lock-up areas.

### Challenges to implementation

Three challenges to the implementation of the APC were described: (1) time (2), engagement, and (3) costs. Having time (and capacity) to deliver PA promotion in primary care was highlighted as a barrier to implementation. Engagement of staff and patients was described as a challenge. Finally, six respondents highlighted costs as a barrier to implementation, with reference mainly to infrastructure costs including standing desks and bike storage. Acknowledging both the potential and barriers to implementation of the APC, one respondent called for more support (e.g., time, signposting) to address barriers and maximise potential:

“[…] great idea, [but] I feel I need more in house support to deliver more effectively” (S2, GP).

## Discussion

### Summary

Despite the exploratory survey indicating a positive outlook on the APC, respondents felt that the potential impact was not being fulfilled. Three major challenges were highlighted – (1) time (2), engagement, and (3) costs. Responses also indicated a need to address these challenges with additional support.

### Comparison with existing literature and implications for practice

The perceived impact of the APC was largely positive. Respondents perceived the APC to have had a positive impact at increasing PA levels (64%, *n* = 21) and decreasing sedentary behaviour in staff (61%, *n* = 20). A small, but noticeable, number of respondents did not feel the APC had a positive impact on either physical activity or sedentary behaviour in patients and staff. Given PHPs’ personal PA levels and perception of PA is one of the major facilitators of PA promotion [14], actions which improve staff PA levels are important and need to be encouraged.

The impact of the APC on sedentary behaviour in patients was less clear. Previous research has failed to address the impact of primary care interventions on sedentary behaviour [8], with a lack of valid and reliable measurements of sedentary behaviour making measurement difficult. Future research is required to objectively measure changes in PA (e.g., pedometers) and sedentary time (e.g., observation of PHPs’ working-time sedentary behaviour) to assess the impact of the APC on behaviour. An improved understanding of patient perspectives would also be beneficial. The free-text responses revealed a more nuanced assessment of impact of the APC. Although many comments echoed the positive findings of the quantitative data, some were less endorsing. One comment – “what is it other than a badge?” – highlighted what appears to be a misconception of the value of the APC and the risk that the award-based system of the APC attracts practices that are already delivering the service. Although staff and patient education are perceived to be major elements of the APC, more education to address this misconception around the value of the APC may be warranted. This is particularly important given the role PHPs’ self-efficacy has as a predictor of likelihood to promote PA [12, 17]. Subsequently two key points need to be illustrated, first the effectiveness of brief interventions at increasing PA in patients [18] as emphasised by the investment of governing bodies (e.g., *World Health Organisation BRIEF toolkit* [19]). And second, the perceived effectiveness of the APC, as highlighted in this study, as a means to deliver PA promotion.

Furthermore, given practitioner behaviour is a strong predictor of their likelihood to promote PA [20, 21], a lack of staff engagement in the APC needs to be addressed. This lack of engagement is multifactorial, with a number of factors being identified in previous research that limit implementation, echoing some of the findings in this study, including: (a) time constraints; (b) insufficient knowledge and skills; (c) a lack of resources and support; and (d) negative financial implications [14, 17]. Addressing all the barriers is largely beyond the remit of this work, with input required at an institutional and/or governmental level (as opposed to just an individual one). However, the APC acts to address a lack of resources and support, whilst also providing education through the online toolkit [15]. Adapting these resources, improving access, and considering a minimum level of mandatory education for accredited practices as part of future APC changes should be considered.

In this survey, PHPs felt that ‘time constraints’ were a major barrier to the implementation of the APC. This is consistent with current literature, with ‘time’ being repeatedly highlighted as a major barrier to the implementation of PA measures in primary care settings [20–43]. Given the broader context of the current GP crisis [44], and the predicted longevity of this issue [45], ingenuity is required to make future interventions place minimal demands on PHPs’ time. One solution is social prescribers with a particular focus on PA, with evidence of increasing uptake in the UK [46]. Policy is recognising this, with a recent Scottish government report recommending that all GP practices employ a PA focussed social prescriber [47]. This should, however, be caveated by the fact that the evidence-base for the effectiveness of social prescribing remains limited [48], and financial support from health bodies and/or governments for such an approach is often lacking.

Cost as a barrier to PA promotion is consistent across research and is supported by the findings in this exploratory study [20, 30, 37, 49]. Financially incentivising PHPs to deliver lifestyle changes has been effective (e.g., smoking cessation) [50] and, given that brief advice for PA is more effective at inducing behaviour change than brief advice for smoking cessation, the benefits of incentives through primary care could be significant [51, 52].

Despite these challenges, one of the themes that was developed from this research was the perceived improvement in staff well-being due to the APC. Given the declining morale in the NHS [44], reversing this is of huge significance. PA-based challenges (e.g., implementing a ‘step-count challenge’) were highlighted repeatedly – specifically related to their function in improving cohesion and morale. This might have implications as to what the RCGP can offer to improve staff well-being in future iterations of the APC initiative.

### Strengths and limitations

This study is novel, with it being the first study (to the best of our knowledge) to assess the impact of any UK wide general practice-based initiative to promote PA. The RCGP are being proactive in the evaluation of the APC to refine and improve implementation – acknowledging that successful implementation is an iterative and context-dependent process.

However, the analysis was performed on a small number of respondents (*n* = 33), with a response rate of 11.4% (*n* = 21) of APC accredited practices in the UK. As a consequence, care should be taken not to generalise or overstate the results. This response rate may have been influenced by unprecedented pressures on PHPs at the time of the survey because of health economic circumstances. These circumstances include a global pandemic (COVID-19) for three years of the evaluation period, which caused a significant shift in not only healthcare delivery but also patient’s health [53]. Given these pressures, response rates and/or responder bias may have been amplified in these results, possibly including more respondents who have a pre-existing interest in PA promotion. Future work should consider utilisation of incentives for participation (e.g., “thank you” payments) and the adoption of a more extensive communication strategy (e.g., social media, digital newsletters) to maximise survey response rate. The results from this exploratory study, however, offer valuable insights to direct future studies into the APC, and identify the need for a longitudinal assessment – without the challenges of a global pandemic – to track changes over time. Although qualitative analysis of free-text responses in this survey helped generate additional insight, it is acknowledged that this is not a comprehensive way to conduct qualitative research (i.e., small q). Therefore, it is recommended that additional qualitative research efforts (i.e., Big Q) are required to build upon the findings from this exploratory study.

## Conclusion

PHPs reported that the RCGP’ APC initiative increased staff PA and had a positive effect on staff integration and morale. The nature of the APC requires practices to prove they have met the pre-stated requirements. This is likely to have an inherent selection bias and fails to address some of the major barriers to PA promotion in primary care – time, engagement, and cost. Consequently, this study highlights a need for further support of primary care teams to deliver PA promotion, with development of the APC initiative required. Future iterations of the APC should seek to take a more proactive approach to address the main barriers to APC promotion – time, finance, and resources – and offer improved support to aid successful implementation.

### Electronic supplementary material

Below is the link to the electronic supplementary material.

Supplementary Material 1

## Data Availability

The dataset underlying this article is not publicly available as the study participants did not give consent for their data to be shared publicly. However, the data are available from the corresponding author on reasonable request.

## Abbreviations

APC: Active practice charter
JOS: Jisc Online Surveys
NHS: National Health Service
PA: Physical activity
PHP: Primary healthcare professionals
RCGP: Royal college of general practitioners

## References

[1] Hardman AE, Stensel DJ (2009). Physical activity and health: the evidence explained.

[2] Foster C (2019). UK Chief Medical officers’ physical activity guidelines.

[3] Bull FC (2016). Start active, stay active.

[4] Department for Health and Social Care (2022). Physical activity: applying all Our Health. Department for Health and Social Care EaW.

[5] McNally S, Exercise (2015). The miracle cure and the role of the doctor in promoting it.

[6] Lion A, Vuillemin A, Thornton JS, Theisen D, Stranges S, Ward M (2019). Physical activity promotion in primary care: a utopian quest?. Health Promot Int.

[7] Haskell WL (2003). Cardiovascular disease prevention and lifestyle interventions: effectiveness and efficacy. J Cardiovasc Nurs.

[8] Kettle VE, Madigan CD, Coombe A, Graham H, Thomas JJC, Chalkley AE (2022). Effectiveness of physical activity interventions delivered or prompted by health professionals in primary care settings: systematic review and meta-analysis of randomised controlled trials. Br J Med.

[9] Campbell F, Holmes M, Everson-Hock E, Davis S, Buckley Woods H, Anokye N (2015). A systematic review and economic evaluation of exercise referral schemes in primary care: a short report. Health Technol Assess.

[10] Coote A. Prevention Rather than Cure. In: King’s Fund, editor. King’s Fund Publications. London, 2004.

[11] WHO (2017). Tackling NCDs best buys.

[12] Chatterjee R, Chapman T, Brannan MG, Varney J (2017). GPs’ knowledge, use, and confidence in national physical activity and health guidelines and tools: a questionnaire-based survey of general practice in England. Br J Gen Pract.

[13] Barnes PM, Schoenborn CA. Trends in adults receiving a recommendation for exercise or other physical activity from a physician or other health professional. NCHS Data Brief. 2012;(86):1–8. 22617014

[14] Leese C, Abraham K, Smith BH. Narrative review – barriers and facilitators to promotion of physical activity in primary care. Lifestyle Med. 2023;n/a(n/a):e81.

[15] RCGP. Physical Activity Hub London: Royal College of General Practitioners. 2018 [Available from: https://elearning.rcgp.org.uk/course/view.php?id=536

[16] Hsieh HF, Shannon SE (2005). Three approaches to qualitative content analysis. Qual Health Res.

[17] Woodhead G, Sivaramakrishnan D, Baker G (2023). Promoting physical activity to patients: a scoping review of the perceptions of doctors in the United Kingdom. Syst Reviews.

[18] Lamming L, Pears S, Mason D, Morton K, Bijker M, Sutton S (2017). What do we know about brief interventions for physical activity that could be delivered in primary care consultations? A systematic review of reviews. Prev Med.

[19] Ferreira-Borges C, Wickramansinghe K, Malykh R, Hetz K, Breda J (2022). Integrated brief interventions for noncommunicable disease risk factors in primary care: the manual. BRIEF project.

[20] Lowe A, Myers A, Quirk H, Blackshaw J, Palanee S, Copeland R. Physical activity promotion by GPs: a cross-sectional survey in England. BJGP Open. 2022;6(3). 10.3399/BJGPO.2021.0227 PMC9680755 35487584

[21] Hébert ET, Caughy MO, Shuval K (2012). Primary care providers’ perceptions of physical activity counselling in a clinical setting: a systematic review. Br J Sports Med.

[22] Lim RBT, Wee WK, For WC, Ananthanarayanan JA, Soh YH, Goh LML (2020). Correlates, facilitators and barriers of physical activity among primary care patients with prediabetes in Singapore - a mixed methods approach. BMC Public Health.

[23] Jones M, Bright P, Hansen L, Ihnatsenka O, Carek PJ (2021). Promoting physical activity in a primary care practice: overcoming the barriers. Am J Lifestyle Med.

[24] Omura JD, Bellissimo MP, Watson KB, Loustalot F, Fulton JE, Carlson SA (2018). Primary care providers’ physical activity counseling and referral practices and barriers for cardiovascular disease prevention. Prev Med.

[25] Leenaars KE, Smit E, Wagemakers A, Molleman GR, Koelen MA (2015). Facilitators and barriers in the collaboration between the primary care and the sport sector in order to promote physical activity: a systematic literature review. Prev Med.

[26] Singer J, Lindsay EA, Wilson DM (1991). Promoting physical activity in primary care: overcoming the barriers. Can Fam Physician.

[27] Keohane D, Mulligan N, Daly B. Physical activity levels and perceived barriers to exercise participation in Irish General Practitioners and General Practice trainees. 2018. 29952439

[28] Carstairs SA, Rogowsky RH, Cunningham KB, Sullivan F, Ozakinci G. Connecting primary care patients to community-based physical activity: a qualitative study of health professional and patient views. BJGP Open. 2020;4(3). 10.3399/bjgpopen20X101100 PMC7465571 32694135

[29] AuYoung M, Linke SE, Pagoto S, Buman MP, Craft LL, Richardson CR (2016). Integrating physical activity in primary care practice. Am J Med.

[30] Leenaars KE, Florisson AM, Smit E, Wagemakers A, Molleman GR, Koelen MA (2016). The connection between the primary care and the physical activity sector: professionals’ perceptions. BMC Public Health.

[31] Hall LH, Thorneloe R, Rodriguez-Lopez R, Grice A, Thorat MA, Bradbury K (2022). Delivering brief physical activity interventions in primary care: a systematic review. Br J Gen Pract.

[32] Douglas F, Torrance N, van Teijlingen E, Meloni S, Kerr A (2006). Primary care staff’s views and experiences related to routinely advising patients about physical activity. A questionnaire survey. BMC Public Health.

[33] Din NU, Moore GF, Murphy S, Wilkinson C, Williams NH (2015). Health professionals’ perspectives on exercise referral and physical activity promotion in primary care: findings from a process evaluation of the National Exercise Referral Scheme in Wales. Health Educ J.

[34] Charles M, Ouchchane L, Thivel D, Celine L, Duclos M (2022). Does legislative framework favors prescription of physical activity in primary care ? The French experience. Phys Sportsmed.

[35] Abramson S, Stein J, Schaufele M, Frates E, Rogan S (2000). Personal exercise habits and counseling practices of primary care physicians: a national survey. Clin J Sport Med.

[36] Leenaars K, Smit E, Wagemakers A, Molleman G, Koelen M (2018). The role of the care sport connector in the Netherlands. Health Promot Int.

[37] Maula A, LaFond N, Orton E, Iliffe S, Audsley S, Vedhara K (2019). Use it or lose it: a qualitative study of the maintenance of physical activity in older adults. BMC Geriatr.

[38] Matoff-Stepp S (2012). Findings and recommendations from the interim evaluation of the bright futures for women’s health and wellness physical activity and healthy eating tools. Health Promot Pract.

[39] Dean S, Elley C, Kerse N. Physical activity promotion in general practice: patient attitudes. Aus Fam Physician. 2007;36(12). 18075637

[40] Hong YA, Forjuoh SN, Ory MG, Reis MD, Sang HA, Multi-Level (2017). Mobile-enabled intervention to promote physical activity in older adults in the primary care setting (iCanFit 2.0): protocol for a Cluster Randomized Controlled Trial. JMIR Res Protoc.

[41] Sherman MD, Hooker SA (2020). Family medicine physicians’ confidence and perceived effectiveness in delivering health behaviour change interventions. Fam Pract.

[42] Dickfos M, King D, Parekh S, Boyle FM, Vandelanotte C (2015). General practitioners’ perceptions of and involvement in health behaviour change: can computer-tailored interventions help?. Prim Health care Res Dev.

[43] Wattanapisit A, Wattanapisit S, Wongsiri S (2021). Overview of physical activity counseling in primary care. Korean J Fam Med.

[44] Gerada C. Clare Gerada: from clap to slap—general practice in crisis. BMJ. 2021;374. 10.1136/bmj.n2224 34521647

[45] Shembavnekar NBJ, Bazeer N, Kelly E, Beech J, Charlesworth A, McConkey R, Fisher R. NHS workforce projections 2022. The Health Foundation2022.

[46] Arie S. The health coaches from Dunkin’Donuts. Br J Med. 2015;350. 10.1136/bmj.h1456 25817137

[47] Martin G, O’Kane P, Callaghan S, Gulhane S, Health SCSC (2022). Tackling health inequalities in Scotland.

[48] Husk K, Elston J, Gradinger F, Callaghan L, Asthana S. Social prescribing: where is the evidence? Brit J Gen Pract. 2019:6–7. 10.3399/bjgp19X700325 PMC6301369 30591594

[49] Josyula LK, Lyle RM (2013). Barriers in the implementation of a physical activity intervention in primary care settings: lessons learned. Health Promot Pract.

[50] Szatkowski L, Aveyard P (2016). Provision of smoking cessation support in UK primary care: impact of the 2012 QOF revision. Br J Gen Pract.

[51] Stead LF, Bergson G, Lancaster T. Physician advice for smoking cessation. Cochrane Database Syst Rev. 2008;(2):Cd000165. 10.1002/14651858.CD000165.pub3 18425860

[52] Orrow G, Kinmonth AL, Sanderson S, Sutton S (2012). Effectiveness of physical activity promotion based in primary care: systematic review and meta-analysis of randomised controlled trials. BMJ.

[53] Brown A, Flint SW, Kalea AZ, O’Kane M, Williams S, Batterham RL. Negative impact of the first COVID-19 lockdown upon health-related behaviours and psychological wellbeing in people living with severe and complex obesity in the UK. EClinicalMedicine. 2021;34. 10.1016/j.eclinm.2021.100796 PMC7970262 33754138

