# Supplementary material for: A movement for movement: an exploratory study of primary healthcare professionals’ perspectives on implementing the Royal College of General Practitioners’ active practice charter initiative
Source: BMC Prim Care. 2024 Apr 15;25:112. doi: 10.1186/s12875-024-02345-0 (PMC11017589; doi:10.1186/s12875-024-02345-0)
Supplement: Supplementary file 1 — Supplementary Material 1 [file 12875_2024_2345_MOESM1_ESM.docx]

Supplementary File 1: Survey Questions

*What is the name of your GP Practice?**

[Free Text]

*What is your role within the practice?**

General Practitioners

GP Trainee

Practice Nurse

Administration Team

Other

*What is the estimated list size?*

<5000

5000-10,000

10,000-15,000

>15,000

*Which community based physical activity provider are you currently affiliated with (or in the process of affiliating with)? Please select one or more if appropriate.**

Parkrun

RunTalkRun

Community Walking Group

Not Applicable

Other (or multiple)

*Since your practice gained Active Practice Charter accreditation have your own (personal) levels of physical activity and/or sedentary behaviour changed?*

Yes/No

## *How has your physical activity and/or sedentary behaviour changed?*

[Free Text]

## *How has being signed up to the 'Active Practice Charter' changed how your practice delivers healthcare, if at all? Where possible, please outline examples of ways healthcare delivery has changed*.*

[Free Text]

*For the following statements please indicate your level of agreement:*

1. Becoming an ‘Active Practice’ has been effective at improving staff physical activity levels

| Strongly agree |
| --- |
| Agree |
| Neither agree nor disagree |
| Disagree |
| Strongly disagree |

1. Becoming an ‘Active Practice’ has been effective at decreasing staff sedentary behaviour

| Strongly agree |
| --- |
| Agree |
| Neither agree nor disagree |
| Disagree |
| Strongly disagree |

1. Becoming an ‘Active Practice’ has been effective at improving patient physical activity levels

| Strongly agree |
| --- |
| Agree |
| Neither agree nor disagree |
| Disagree |
| Strongly disagree |

1. Becoming an ‘Active Practice’ has been effective at decreasing patient sedentary behaviour

| Strongly agree |
| --- |
| Agree |
| Neither agree nor disagree |
| Disagree |
| Strongly disagree |

## *If willing, please provide any justification/evidence for your answers.*

[Free Text]

## *What have been the biggest challenges to implementing the ‘Active Practice Charter’ within your practice? Please provide at least two examples*.*

[Free Text]

## *What positive changes have resulted from becoming an 'Active Practice'? Please provide at least two examples*.*

[Free Text]

## *Do you have any general comments about the ‘Active Practice Charter’?*

[Free Text]
